# Effects of participatory learning and action with women’s groups, counselling through home visits and crèches on undernutrition among children under three years in eastern India: a quasi-experimental study

**DOI:** 10.1186/s12889-019-7274-3

**Published:** 2019-07-18

**Authors:** Raj Kumar Gope, Prasanta Tripathy, Vandana Prasad, Hemanta Pradhan, Rajesh Kumar Sinha, Ranjan Panda, Jayeeta Chowdhury, Ganapathy Murugan, Shampa Roy, Megha De, Sanjib Kumar Ghosh, Swati Sarbani Roy, Audrey Prost

**Affiliations:** 1 Ekjut, Chakradharpur, Jharkhand India; 2 Public Health Resource Society, New Delhi, India; 3 Child In Need Institute (CINI), Pailan, West Bengal India; 4 Tata Trusts, Mumbai, Maharashtra India; 5 Institute for Global Health, University College London, London, UK

**Keywords:** Community, Community mobilisation, Child undernutrition, India, South Asia, Groups, Home visits, Crèche, Day care, Quasi-experimental

## Abstract

**Background:**

India faces a high burden of child undernutrition. We evaluated the effects of two community strategies to reduce undernutrition among children under 3 years in rural Jharkhand and Odisha, eastern India: (1) monthly Participatory Learning and Action (PLA) meetings with women’s groups followed by home visits; (2) crèches for children aged 6 months to 3 years combined with monthly PLA meetings and home visits.

**Methods:**

We tested these strategies in a non-randomised, controlled study with baseline and endline cross-sectional surveys. We purposively selected five blocks of Jharkhand and Odisha, and divided each block into three areas. Area 1 served as control. In Area 2, trained local female workers facilitated PLA meetings and offered counselling to mothers of children under three at home. In Area 3, workers facilitated PLA meetings, did home visits, and crèches with food and growth monitoring were opened for children aged 6 months to 3 years. We did a census across all study areas and randomly sampled 4668 children under three and their mothers for interview and anthropometry at baseline and endline. The evaluation’s primary outcome was wasting among children under three in areas 2 and 3 compared with area 1, adjusted for baseline differences between areas. Other outcomes included underweight, stunting, preventive and care-seeking practices for children.

**Results:**

We interviewed 83% (3868/4668) of mothers of children under three sampled at baseline, and 76% (3563/4668) at endline. In area 2 (PLA and home visits), wasting among children under three was reduced by 34% (adjusted Odds Ratio [aOR]: 0.66, 95%: 0.51–0.88) and underweight by 25% (aOR: 0.75, 95% CI: 0.59–0.95), with no change in stunting (aOR: 1.23, 95% CI: 0.96–1.57). In area 3, (PLA, home visits, crèches), wasting was reduced by 27% (aOR: 0.73, 95% CI: 0.55–0.97), underweight by 40% (aOR: 0.60, 95% CI: 0.47–0.75), and stunting by 27% (aOR: 0.73, 95% CI: 0.57–0.93).

**Conclusions:**

Crèches, PLA meetings and home visits reduced undernutrition among children under three in rural eastern India. These interventions could be scaled up through government plans to strengthen home visits and community mobilisation with Accredited Social Health Activists, and through efforts to promote crèches.

**Trial registration:**

The evaluation was registered retrospectively with Current Controlled Trials as ISCRTN89911047 on 30/01/2019.

**Electronic supplementary material:**

The online version of this article (10.1186/s12889-019-7274-3) contains supplementary material, which is available to authorized users.

## Background

In India, 38% of children under five are stunted and 21% are wasted. [1] Several nutrition-specific interventions are recommended to reduce this burden. These include increasing access to diverse foods for girls and women, delaying the first pregnancy, providing antenatal care and iron supplementation, appropriate infant and young child feeding (IYCF), preventive actions and care-seeking for childhood illnesses, and treatment for children with Severe Acute Malnutrition (SAM). [2] It is also essential to support nutrition-sensitive interventions including women’s education and empowerment, safe water, sanitation, and sustainable livelihoods. [3] Several nutrition-specific interventions are supported by frontline workers from the Ministry of Women and Child Development (*Anganwadi* workers) and the Ministry of Health and Family Welfare (Accredited Social Health Activists, or ASHAs). Unfortunately, levels of child undernutrition remain high despite these workers’ efforts, especially among underserved communities. Children from Scheduled Tribe families, in particular, have the highest prevalence of stunting in the country (44%). [1] There is a critical need for research focused on *how* to deliver nutrition interventions in such underserved communities.

In this study, we report results from Action Against Malnutrition (AAM), a civil society-led, community-based initiative to supplement the efforts of frontline health and nutrition workers in seven blocks (administrative sub-divisions of 60,000–120,000 population) of Jharkhand, Odisha, Bihar and Chhattisgarh. AAM included two community strategies to reduce undernutrition among children under 3 years: monthly Participatory Learning and Action (PLA) meetings with women’s groups followed by counselling through home visits; (2) crèches for children aged 6 months to 3 years combined with PLA meetings and home visits. We evaluated the effect of these two strategies on child wasting, underweight, stunting, infant and young child feeding, illness and care during illness, as well as infection control practices.

## Methods

### Study setting

The AAM initiative was implemented between July 2012 and March 2017. It covered seven blocks in seven districts of four states: three blocks in Jharkhand, two in Odisha, one in Chhattisgarh, and one in Bihar. These four states have high levels of chronic child undernutrition: 45% of children in Jharkhand are stunted, 34% in Odisha, 38% in Chhattisgarh and 48% in Bihar. The prevalence of acute undernutrition is also high: 29% of children in Jharkhand are wasted, 20% in Odisha, 23% in Chhattisgarh and 21% in Bihar. In addition, several districts of Jharkhand, Odisha and Chhattisgarh have a high proportion of families from *Adivasi* (indigenous, or Scheduled Tribe) communities disproportionately affected by undernutrition [1, 4, 5].

### Interventions

AAM supported one community-based facilitator to conduct Participatory Learning and Action (PLA) meetings and home visits in a catchment area of approximately 5000 population, and two workers for crèches open to children aged 6 months to 3 years.

#### PLA meetings and counselling through home visits

The PLA intervention was a structured cycle of participatory women’s groups meetings convened by a local female facilitator. The cycle of meetings had four phases. In the first phase, women identified and prioritised problems related to undernutrition among children under three (e.g. lack of dietary diversity, diarrhoea, malaria) using picture cards. They then identified the underlying medical and social causes for these prioritised problems through story-telling and discussions, before prioritising locally feasible strategies to address these problems. At the end of this phase, each group organised a meeting with the wider community to seek support for its chosen strategies. In the third phase, groups implemented their strategies and learned about practical actions to try at home and in the community (e.g. methods to enrich complementary foods or clearing stagnant water ponds). In the fourth phase, each group evaluated the meeting cycle. This intervention was an adaptation of an approach previously tested in Jharkhand and Odisha to improve birth outcomes. [6–8] Similar PLA meeting cycles have since been developed and scaled up to improve child health and nutrition in Odisha and Bihar [9, 10].

The PLA facilitator also provided counselling to mothers of children under three through home visits. We chose to do home visits because a concurrent study suggested that mothers appreciated the additional conversations, demonstrations and follow-up after PLA meetings, and because home visits are recommended in World Health Organisation (WHO) tools for the Integrated Management of Childhood Illness and Infant and Young Child Feeding counselling [11, 12]. During home visits, facilitators and mothers discussed early and exclusive breastfeeding, timely initiation of complementary feeding, how to enrich complementary foods, danger signs related to childhood illnesses, preventive measures (handwashing and the use of bed nets), and the importance of seeking care from trained providers. During PLA meetings, the facilitator used picture cards, story-telling, role plays, games, and demonstrations of handwashing and food enrichment. During the home visits, they used MUAC tapes, a pictorial counselling tool and the Mother and Child Protection (MCP) cards for age-appropriate counselling. The facilitators’ visits were expected to cover approximately 10–15% of homes with children under three in a given month. Facilitators prioritised visits to the following children under three years: those identified as having MUAC< 11.5 cm or as severely underweight during PLA meetings; those identified as underweight by *Anganwadi* workers; those who recently had an illness; those who lived in hamlets; and those who had recently been discharged from a Malnutrition Treatment Centre or Nutritional Rehabilitation Centre. All facilitators received a total of 12 days of training on the PLA meeting cycle and counselling. They subsequently had fortnightly review meetings with supervisors (one supervisor for 15 facilitators), during which they discussed problems and solutions to issues encountered during counselling. Each facilitator received an incentive of 3500 Indian rupees (US $ 60) per month, i.e. a little more than the 3000 INR salary paid to *Anganwadi* workers at the time. She was responsible for conducting 8–10 monthly PLA meetings as well as visiting 25–35 mothers of children under three in an area covering around 5000 population.

#### Crèches for children aged 6 months to 3 years

AAM selected crèches as an additional, more intensive intervention to prevent undernutrition because they offered the possibility of co-locating several services: free care in a safe, smoke-free environment with clean drinking water, handwashing stations, nutritious food, growth monitoring, and psychosocial stimulation.

Children in crèches received one full meal and two snacks per day, as well as two eggs per week. Meal and snack preparation were supervised to ensure caloric and protein sufficiency. The programme was available to every child in the village irrespective of their nutritional status. Within this ‘universe’ however, a special focus was given to crèche-going children who did not gain weight or whose weight decreased over two consecutive months, our operational definition of growth faltering. Children whose growth faltered and those with severe underweight were given two additional calorie-dense and protein rich meals per day. We also developed a protocol for persistent growth faltering which involved home visits and medical referrals, as described in more detail elsewhere [13].

Each crèche was run by two local, trained workers who were not *Anganwadi* workers. Like PLA facilitators, crèche workers received 3500 INR per month. Community members participated in the selection of workers, decided on opening hours and designed the food menu. They also helped fence, repair, and decorate crèches, took part in crèche committee meetings, and sometimes contributed vegetables from their homestead gardens. In four villages, community members built the crèches themselves.

### Study design

We conducted a non-randomised, controlled (quasi-experimental) study with cross-sectional baseline and endline surveys to evaluate the effects of the two incrementally intensive strategies on child wasting, underweight, stunting, as well as infant and young child feeding, illness and infection control outcomes. The AAM consortium partners selected five blocks out of the seven participating in the programme for the evaluation: three blocks in Jharkhand (Gola, Khuntpani and Ratu-Nagri) and two in Odisha (Thakurmunda and Saharpada). The block from Bihar was excluded because ensuring adequate separation between intervention and control areas proved too difficult. The block from Chhattisgarh was excluded as crèches were scattered across too large an area, making data collection highly challenging.

To minimise the bias caused by geographical variability, we sought to compare the effects of interventions between areas within the same blocks. Each block was divided into three zones designated as areas 1, 2 and 3. Area 1 covered approximately 20% of each block and received regular government programmes without any intervention from AAM. For the purpose of the evaluation, area 1 was therefore designated as a control area. Area 2 covered approximately 65% of each block and received PLA meetings and counselling through home visits. Area 3 covered approximately 15% of each block and received PLA meetings, counselling through home visits, and crèches. AAM started a total of 116 crèches across the five blocks: 40 in Odisha and 76 in Jharkhand. AAM partners deliberately selected areas that had a high proportion of tribal families to open crèches in order to provide the greatest inputs to the most underserved.

**Fig. 1:**
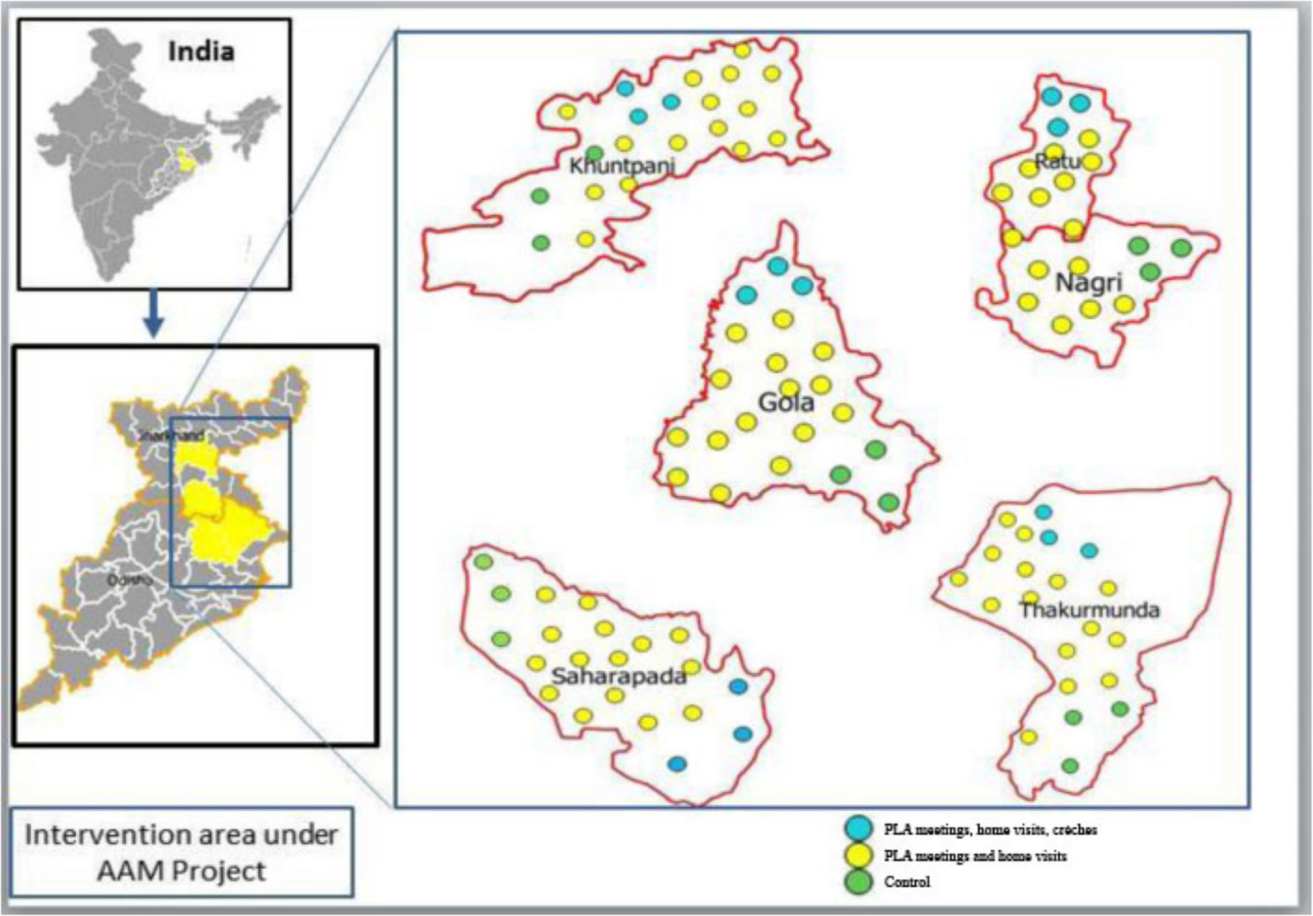
Location of study areas (Wikemedia and authors’ own)

**Fig. 2:**
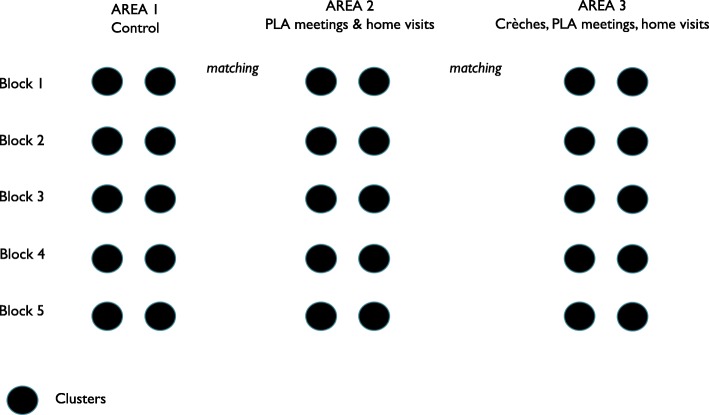
Study design (authors’ own)

Within each of the five evaluation blocks, we mapped population clusters of 8–10 contiguous villages (c.4000–5000 population each). Figure 1 describes the location of blocks and clusters, and Fig. 2 describes the study design. We matched groups of two clusters in each area so that each block had comparable clusters across all three areas, as described in Fig. 2. The matching criteria were population size, percentage of Scheduled Tribe and Scheduled Caste population, number of *Anganwadi* centres, and number of health sub-centres. Using this method, a total of 30 population clusters were selected from the five evaluation blocks, or six clusters per block: 10 in Area 1, 10 in Area 2 and 10 in Area 3. The estimated total population in the three evaluation areas was 144,000.

### Participants

Study participants were all children under 3 years and their mothers identified during a household census in the study clusters, then selected via simple random sampling as described in the sampling section below. There were no exclusion criteria.

### Outcomes

The primary outcome for the evaluation was wasting among children under 3 years, as we expected interventions primarily focused on immediate and underlying determinants of undernutrition to have a larger effect on acute rather than chronic undernutrition. Secondary outcomes included underweight, stunting, Infant and Young Child Feeding (IYCF) practices, infection control practices, and uptake of nutrition and health services.

### Sample size

We estimated the baseline prevalence of wasting in the study areas at 23% using district-level data from the Hungama study [14]. We hypothesised a 2% point prevalence ‘secular’ reduction in wasting over the course of the evaluation in area 1 (from 23 to 21%), a 7% point reduction in area 2 following the interventions (from 23 to 16%), and a 12% point reduction in area 3 (from 23 to 11%). To detect the smallest difference in wasting across areas in the endline survey (a 5% difference in wasting between area 1 and 2), we required 4668 children aged 0–36 months (1556 per area) with a 10% refusal rate and a design effect of 1.5 to account for clustering.

We used the 2011 Indian Census and Crude Birth Rate data from the Annual Health Survey (2011) for Jharkhand and Odisha to estimate the total number of children under three across the three study areas. The CBR for Jharkhand and Odisha were 25 and 23; we used a conservative estimate of 22 and estimated that we would find 9504 children under 3 years across all three study areas. To reach our target sample size of 4668 children, we sampled one out of two children under three in the study areas. For both the baseline and endline surveys, we first carried out a household census in all clusters to identify all children under three, then used simple random sampling to select children for anthropometric measurements.

### Data collection

We conducted the cross-sectional baseline survey from October to March 2012–13 and the endline survey from October to April, 2015–16. Additional file 1 is the questionnaire used in both surveys. Prior to each survey, we organised 4 days of training in each district, including 1 day of training on anthropometry and a day of practice with children under three in a community setting. After two measurements for 10 children, data collectors with differences greater than 0.7 cm in height and 0.5 mm for MUAC between the two readings were given additional training and practical tests. During data collection, supervisors observed 5% of all interviews and measurements. We used Equinox weighing scales with 100 g graduations, calibrated twice a week to measure weight. We used infantometers (Seca 210 with 5 mm graduation) and stadiometers (Seca 213 with 1 mm graduation) for length/height measurements. In each of the five evaluation blocks, nine locally recruited interviewers collected data with support from one coordinator and one program officer.

### Cost data

We collected data on the costs of all AAM interventions from each of the implementing agencies that supported them on an annual basis through a standardised tool. All costs were adjusted for inflation, discounted at 3% per year and converted to 2017 United States Dollars (USD).

### Data management and statistical analysis

Data were entered in a MS-Access database in each district, then cleaned using checks on range and frequencies. We used descriptive statistics to explore the socio-demographic characteristics of women and children and the macro z score in Stata 13.0 to generate z scores for anthropometry [15]. In addition to anthropometric primary and secondary outcomes defined a priori, we generated a Composite Index of Anthropometric Failure coded as 1 if a child was wasted, underweight, stunted or any combination of these, and 0 if they were not wasted, underweight or stunted [16]. This was intended to provide a more global measure of undernutrition.

Analyses were by intention to treat, meaning that children were included in the analyses if they participated in baseline or endline surveys, whether they took part in the interventions or not. We used logistic regression with random effects at cluster-level in Stata 13.0 to analyse data on binary outcomes (e.g. wasting), and generalised estimating equations (GEE) with an exchangeable correlation for continuous outcomes (e.g. weight-for-height z scores). We decided, a priori*,* to adjust all analyses for district, tribe/caste status, asset quintiles and clustering. We created these asset quintiles using Principal Component Analysis. We also selected additional variables for adjustment by identifying household, maternal and child characteristics that differed significantly (*p* < 0.05) between areas at baseline and endline. We checked how strongly these factors were associated with each other and dropped two of them (agricultural land ownership and source of staple food) because they were collinear with each other and with asset quintiles. We estimated intervention effects using the difference in difference method, and effect sizes are presented as adjusted odds ratios. We adjusted for baseline differences by introducing an interaction term between area and survey wave (baseline or endline) in logistic regression models.

### Ethical approval

We obtained ethical approval for the study from the Institutional Ethics Committee of the Public Health Resource Network (PHRN) in Delhi on the 14th of February 2012, and from an independent ethics committee convened by Ekjut in Ranchi (Jharkhand) on the 10th of May 2013. The survey team requested consent for participation in the survey and anthropometry from mothers. This was recorded in writing or through a thumbprint impression.

## Results

In the baseline survey, we found and measured 88% (1365/1556) of our target sample of children under 3 years in area 1, 80% (1248/1556) in area 2, and 68% (1255/1556) in area 3. In the endline survey, we measured 75% (1168/1556) of our target sample in area 1, 76% (1255/1556) in area 2, and 73% (1140/1556) in area 3.

Table 1 describes the socio-demographic characteristics of households, mothers and children in baseline and endline surveys. We found differences in households’ ownership of agricultural land, assets, and Mahatma Gandhi National Rural Employment Guarantee Act (MNREGA) job card, as well as in tribe/caste status and maternal education at both baseline and endline. Over three quarters of households had agricultural land, with some differences between areas (*p*-value for differences between areas < 0.001). Between 48 and 71% of families had MNREGA job cards (*p* < 0.001) that guaranteed 100 days of waged employment for every household whose adult members volunteered to do unskilled manual work. The proportion of mothers from Scheduled Tribes ranged from 61% in area 1 (control) to 83% in area 3 (*p* < 0.001). Between 54 and 60% of mothers had ever been to school (*p* < 0.001). We found differences in the mean ages of children who participated in the survey between areas at baseline (*p* = 0.017) but none at endline (*p* = 0.242).

**Table 1:** Characteristics of households, mothers and children at baseline and endline

	Area 1: Control		Area 2: PLA and home visits		Area 3: Crèches, PLA and home visits		*p* value for differences between areas baseline	*p* value for differences between areas at endline
	Baseline	Endline	Baseline	Endline	Baseline	Endline		
	*N* = 1263	*N* = 1130	*N* = 1245	*N* = 1256	*N* = 1360	*N* = 1177		
Household characteristics, n (%)								
Household owns agricultural land	987 (78.2)	912 (80.7)	1117 (89.7)	1112 (88.5)	1271 (93.4)	1077 (91.5)	< 0.001	< 0.001
Household wealth quintile, n (%)								
Richest	295 (23.4)	281 (24.9)	213 (17.1)	286 (22.8)	226 (16.6)	181 (15.4)	< 0.001	< 0.001
Second richest	203 (16.1)	221 (19.6)	253 (20.3)	270 (21.5)	270 (19.9)	255 (21.7)		
Middle	197 (15.6)	204 (18.1)	251 (20.2)	277 (22.1)	255 (18.8)	304 (25.8)		
Second poorest	249 (19.7)	143 (12.7)	232 (18.6)	219 (17.4)	281 (20.7)	248 (21.1)		
Poorest	319 (25.3)	281 (24.9)	296 (23.8)	204 (16.4)	328 (24.1)	189 (16.1)		
Has improved source of drinking water, n (%) ^a^	906 (71.7)	849 (75.1)	918 (73.7)	911 (72.5)	968 (71.2)	863 (73.3)	0.316	0.341
Main source of family’s staple food, n (%)								
Own production	820 (64.9)	532 (47.1)	884 (71.0)	730 (58.1)	908 (66.8)	714 (60.7)	0.001	< 0.001
Purchased from regular shop	368 (29.1)	420 (37.2)	301 (24.2)	420 (33.4)	352 (25.9)	321 (27.3)		
Purchased from ration shop	73 (5.8)	174 (15.3)	53 (4.3)	95 (7.6)	95 (7.6)	137 (11.6)		
Borrowed, bartered, exchanged	2 (0.2)	4 (0.3)	7 (0.6)	11 (0.9)	4 (0.3)	5 (0.4)		
Household has a MNREGA^b^ job card	687 (54.4)	539 (47.7)	800 (64.3)	755 (60.1)	972 (71.5)	756 (64.2)	< 0.001	< 0.001
Maternal characteristics								
Tribe or Caste, n (%)								
Scheduled Tribe	774 (61.3)	757 (67.0)	857 (68.8)	942 (75.1)	1128 (82.9)	978 (83.1)	< 0.001	< 0.001
Scheduled Caste	57 (4.5)	33 (2.9)	34 (2.7)	21 (1.7)	62 (4.5)	37 (3.1)		
Other Backward Caste	407 (32.2)	322 (28.5)	337 (27.1)	291 (23.2)	161 (11.8)	159 (13.5)		
None of the above	25 (2.0)	18 (1.6)	17 (1.4)	1 (0.1)	9 (0.7)	3 (0.3)		
Mother has ever been to school	722 (57.2)	684 (60.5)	672 (54.0)	683 (54.4)	736 (54.1)	593 (50.4)	0.188	< 0.001
Child characteristics								
Age in months, mean (SD)	20.0 (9.2)	19.0 (9.3)	20.0 (9.8)	18.4 (9.2)	19.0 (9.5)	18.9 (9.4)	0.017	0.242
Sex, n (%)								
Female	615 (45.2)	579 (49.2)	608 (48.8)	607 (48.3)	615 (48.7)	533 (47.2)		
Male	745 (54.8)	598 (50.8)	637 (51.2)	597 (52.8)	648 (51.3)	597 (52.8)	0.108	0.621

^a^ Tubewell or handpump

^b^ Mahatma Gandhi National Rural Employment Guarantee Act

We measured exposure to the interventions: 55% (653/1177) of children in area 3 at endline had ever attended a crèche. Sixty-five percent (815/1256) of mothers of children under three had ever attended a PLA meeting in area 2, as had 72% (853/1177) of mothers in area 3.

### Effects on children’s nutritional status

Table 2 describes effects of the two intervention strategies on wasting, stunting and underweight at endline, adjusted for baseline differences in anthropometry, tribal/caste status, ownership of MNREGA card, maternal education, asset quintile, district and clustering. In area 2, the odds of wasting were reduced by 34% (aOR: 0.66, 95% CI: 0.51–0.88, *p* = 0.004) and the odds of underweight by 25% (aOR: 0.75, 95% CI: 0.59–0.95, *p* = 0.018), with no change in stunting (aOR: 1.23, 95% CI: 0.96-1.57, *p* = 0.099) compared to control. In area 3 (crèches, PLA and home visits), the odds of wasting were reduced by 27% (aOR: 0.73, 95% CI: 0.55–0.97, *p* = 0.028), the odds of underweight by 40% (aOR: 0.60, 95% CI: 0.47–0.75, *p* < 0.001), and the odds of stunting by 27% (aOR: 0.73, 95% CI: 0.57–0.93, *p* = 0.012) when compared to area 1 (control). Using a composite index of anthropometric failure (being wasted, underweight, stunted or any combination of these) we found that children in Area 3 had a reduced odds of anthropometric failure (aOR: 0.61; 0.46–0.79, *p* < 0.001), but not children in Area 2 (aOR: 0.98, 95% CI: 0.75–1.28), *p* = 0.617) [16].

**Table 2:** Effect of the interventions on wasting, underweight, stunting and a composite index of anthropometric failure

	Area 1: Control	Area 2: PLA and home visits	Area 3: Crèches, PLA and home visits	Effect of PLA and home visits vs. control Adjusted OR (95% CI)^a^	*p*	Effect of crèches, PLA and home visits vs control Adjusted OR (95% CI)^a^	*p*
Wasting							
Baseline, N	1214	1190	1315				
n (%)	280 (23.1)	327 (27.5)	314 (23.9)				
Endline, N	1048	1201	1147				
n (%)	282 (26.9)	277 (23.1)	249 (21.7)	0.66 (0.51-0.88)	0.004	0.73 (0.55-0.97)	0.028
Underweight							
Baseline, N	1241	1219	1335				
n (%)	639 (51.5)	644 (52.8)	740 (55.4)				
Endline	1100	1235	1164				
n (%)	603 (54.8)	606 (49.1)	537 (46.1)	0.75 (0.59-0.95)	0.018	0.60 (0.47-0.75)	< 0.001
Stunting							
Baseline (N)	1182	1171	1265				
n (%)	658 (55.7)	594 (50.7)	773 (61.1)				
Endline (N)	1045	1213	1148				
n (%)	564 (54.0)	653 (53.8)	601 (52.3)	1.23 (0.96-1.57)	0.099	0.73 (0.57-0.93)	0.012
Composite index of anthropometric failure^b^							
Baseline	1169	1148	1249				
n (%)	804 (68.8)	789 (68.7)	920 (73.7)				
Endline	993	1169	1126				
n (%)	696 (70.1)	812 (69.5)	733 (65.1)	0.98 (0.75-1.28)	0.617	0.61 (0.46-0.79)	< 0.001

^a^ Differences in wasting, underweight and wasting at endline, adjusted for baseline differences in anthropometry, tribal/caste status (categorical), ownership of MNREGA card (binary), any maternal education (binary), asset quintile (categorical), district (categorical, fixed effect) and clustering (random effect)

^b^The Composite Index of Anthropometric Failure includes six categories of undernutrition: wasting only, wasting and underweight, wasting, stunting and underweight, stunting and underweight, stunting only, underweight only. A child has anthropometric failure if they are either wasted, or underweight or stunted, or any combination of anthropometric failure

We conducted additional sensitivity analyses on continuous z scores to check whether the interventions’ effects on anthropometry were present for weight-for-age (WAZ), weight-for-height (WHZ), and height-for-age (HAZ) z scores. These analyses are presented in Additional file 2. Both WAZ and HAZ scores increased in Area 3 (β: 0.18, 95% CI: 0.02, 0.33 and β: 0.25, 95% CI: 0.05, 0.46, respectively), but we found no effects on WHZ (β: -0.08, 95% CI: − 0.24,0.09). We detected a small, non-significant increase in WHZ and WAZ in Area 2 (β: 0.15, 95% CI: − 0.017, 0.31 and β: 0.14, 95% CI: − 0.012-0.30), and no effect on HAZ (β:-0.11, 95% CI: − 0.31-0.09).

### Effects on the nutritional status of the most marginalised children

Table 3 presents the results of a pre-planned sub-group analysis for the effect of interventions on the nutritional status of children from marginalised families, defined as those belonging to Scheduled Tribes and the two poorest wealth quintiles. In both areas 2 and 3, interventions had strong effects on wasting among children from the most marginalised families when compared to the most marginalised children in area 1 (aOR: 0.53, 95% CI: 0.34–0.82 and aOR: 0.44, 95% CI: 0.28–0.69, respectively).

**Table 3:** Prevalence of wasting at baseline and endline by area and marginalisation status

	Area 1: Control	Area 2: PLA and home visits	Area 3: Crèches, PLA and home visits	Effect of PLA and home visits vs control (adjusted OR^b^)	*p*	Effect of crèches, PLA and home visits vs control (adjusted OR^b^)	*p*
Baseline	1214	1190	1315				
All children	280 (23.1)	327 (27.5)	314 (23.9)				
Most marginalised^a^	114 (25.0)	127 (28.9)	152 (29.1)				
Less marginalised	166 (21.9)	200 (26.7)	162 (20.4)				
Endline	1048	1201	1147				
All children	282 (26.9)	277 (23.1)	249 (21.7)				
Most marginalised	115 (33.0)	89 (23.7)	82 (21.0)	0.53 (0.34-0.82)	0.005	0.44 (0.28-0.69)	< 0.001^1^
Less marginalised	167 (23.9)	188 (22.8)	167 (22.1)	0.73 (0.52-1.03)	0.074	1.01 (0.71-1.43)	0.972

^a^ Most marginalised defined as belonging to tribal families from the two poorest wealth quintiles

^b^ Adjusted for baseline differences in wasting, child age in months (continuous), tribal/caste status (categorical), ownership of MNREGA card (binary), any maternal education (binary), asset quintile (categorical), district (categorical, fixed effect) and clustering (random effect)

^1^ Exact *p* = 0.003

### Infant and young child feeding, infection control and uptake of nutrition services

Table 4 describes effects on infant and young child feeding practices, infection control practices, and the uptake of nutrition services. In area 2, we found improvements in early initiation of breastfeeding, minimum dietary diversity, and the proportion of children consuming iron-rich foods. We also found increases in handwashing before feeding a child and after going the toilet, and the proportion of children sleeping under bed nets and receiving Vitamin A and deworming. Children in Area 2 were also more likely to get ORS in case of diarrhoea, measles immunisations, and to have caregivers who sought advice in case of diarrhoea, fever or cough. In area 3, all the above indicators also increased except for receipt of Vitamin A and deworming.

**Table 4:** Intervention effects on infant and young child feeding, infection control, care-seeking practices, and uptake of services

Survey	Baseline			Endline			Effect of PLA and homevisits vs control Adjusted OR (95% CI) ^a^	*p*	Effect of crèches, PLA and home visits vs control Adjusted OR (95% CI) ^a^	*p*
Areas	1	2	3	1	2	3				
Infant And Young Child Feeding Practices, n (%)										
Children 0-24 months	836	759	854	750	862	782				
Initiation of breastfeeding with 1 h of birth	297 (35.5)	257 (33.9)	324 (37.9)	196 (26.1)	361 (41.9)	370 (47.3)	3.40 (2.41-4.79)	< 0.001	3.80 (2.68-5.38)	< 0.001
Children 12-16 months	166	140	162	136	161	157				
Continued breastfeeding at 1 year	158 (95.2)	135 (96.4)	145 (89.5)	133 (97.8)	150 (93.2)	156 (99.4)	0.08 (0.00-1.01)	0.050	3.40 (0.18-87.5)	0.378
Children 6-9 months	96	99	93	99	129	121				
Timely introduction of semi-solid and solid foods	63 (65.6)	65 (65.7)	66 (71.0)	68 (68.7)	104 (80.6)	105 (86.8)	1.69 (0.64-4.48)	0.292	2.43 (0.86-6.85)	0.093
Minimum meal frequency (breastfed children, twice a day)	55 (57.3)	55 (55.6)	51 (54.8)	59 (59.7)	72 (55.8)	67 (55.4)	0.70 (0.20-1.73)	0.450	0.93 (0.38-2.29)	0.874
Minimum acceptable diet	20 (20.8)	13 (13.3)	25 (26.9)	8 (8.1)	22 (17.0)	38 (31.4)	4.30 (1.26-16.21)	0.019	4.02 (1.30-12.47)	0.016
Children 6-24 months	714	650	756	636	735	684				
Minimum dietary diversity (≥4 food groups)	300 (42.0)	218 (33.5)	352 (46.6)	248 (39.0)	309 (42.0)	474 (69.3)	1.85 (1.31-2.62)	< 0.001	3.67 (2.59-5.21)	< 0.001
Consumption of iron rich foods	245 (34.3)	116 (17.8)	185 (24.5)	193 (30.3)	187 (25.4)	357 (52.2)	2.22 (1.52-3.23)	< 0.001	5.18 (3.64-7.58)	< 0.001
Children 9-24 months	618	551	663	537	606	563				
Minimum meal frequency (breastfed children (three times a day)	402 (65.0)	344 (62.4)	288 (43.4)	371 (69.1)	374 (61.7)	327 (58.1)	0.70 (0.48-1.02)	0.063	1.41 (0.98-2.04)	0.062
Minimum acceptable diet	205 (33.2)	148 (26.9)	161 (24.3)	179 (33.3)	204 (33.7)	228 (40.5)	1.94 (0.91-1.97)	0.135	2.18 (1.50-3.19)	< 0.001
Water, Sanitation And Hygiene Practices, n (%)										
Children 0-36 months	1263	1245	1360	1130	1256	1177				
Water treatment, physical or chemical	288 (22.8)	277 (22.2)	377 (27.7)	326 (28.8)	401 (31.9)	497 (42.2)	1.33 (0.95-1.86)	0.093	1.86 (1.33-2.61)	< 0.001
Handwashing with soap after all three key events^a^	172 (13.6)	48 (3.9)	87 (6.4)	137 (12.1)	245 (19.5)	238 (20.2)	13.1 (8.17-21.2)	< 0.001	8.34 (5.40-12.9)	< 0.001
Preventive Health Practices, Morbidity And Care-Seeking, n (%)										
Children 0-36 months	1263	1245	1360	1130	1256	1177				
Household owns a bed net	955 (75.6)	745 (59.8)	816 (60.0)	754 (66.7)	886 (70.5)	841 (71.5)	3.95 (2.84-5.48)	< 0.001	4.71 (3.40-6.51)	< 0.001
Child slept under a bed net last night (among those in households with a net)	923 (96.7)	513 (68.9)	604 (74.0)	625 (82.9)	790 (89.2)	718 (85.4)	29.1 (16.9.0-50.1)	< 0.001	12.5 (7.29-21.48)	< 0.001
Vitamin A dose received in last 6 months	687 (54.4)	595 (47.8)	888 (65.3)	414 (36.6)	584 (46.5)	527 (44.8)	2.55 (1.96-3.33)	< 0.001	1.15 (0.88-1.49)	0.307
Deworming in last 6 months	626 (49.6)	468 (37.6)	737 (54.2)	361 (32.0)	471 (37.5)	427 (36.3)	2.38 (1.83-3.11)	< 0.001	1.18 (0.91-1.54)	0.205
Diarrhoea in last 14 days	168 (13.3)	166 (13.3)	266 (19.6)	126 (11.2)	165 (13.1)	228 (19.4)	1.30 (0.91-1.86)	0.151	1.22 (0.87-1.72)	0.238
Same or more breastmilk given during diarrhea	84 (50.0)	107 (64.5)	185 (69.5)	105 (83.3)	125 (75.8)	175 (76.7)	0.30 (0.13-0.66)	0.003	0.30 (0.14-0.64)	0.002
Same or more food given during diarrhea	72 (42.9)	80 (48.2)	144 (54.1)	75 (59.5)	101 (61.2)	133 (58.3)	0.72 (0.34-1.53)	0.402	0.88 (0.44-1.74)	0.712
Advice sought from ANM, AWW or ASHA for diarrhea	118 (70.2)	68 (41.0)	117 (44.0)	73 (57.9)	118 (71.5)	178 (78.1)	6.18 (2.92-13.1)	< 0.001	9.82 (4.82-20.0)	< 0.001
ORS used during diarrhea	83 (49.4)	66 (39.8)	103 (38.7)	46 (36.5)	98 (59.4)	129 (56.6)	3.27 (1.52-7.04)	0.002	4.92 (2.39-10.1)	< 0.001
Fever or cough in last 14 days	215 (17.0)	249 (20.0)	407 (29.9)	193 (17.1)	262 (20.9)	428 (36.4)	1.09 (0.79-1.49)	0.595	1.46 (1.08-1.97)	0.013
Same or more breastmilk given in case of fever or cough	135 (62.8)	173 (69.5)	256 (62.9)	152 (78.8)	187 (71.4)	261 (61.0)	0.42 (0.22-0.81)	0.001	0.33 (0.18-0.61)	< 0.001
Same or more food given in case of fever of cough	112 (52.1)	137 (55.0)	172 (42.3)	89 (46.1)	155 (59.2)	176 (41.1)	1.31 (0.73-2.35)	0.363	1.20 (0.71-2.04)	0.488
Advice sought for fever or cough	166 (77.2)	79 (31.7)	199 (48.9)	107 (55.4)	165 (63.0)	240 (56.1)	15.5 (8.12-29.69)	< 0.001	4.44 (2.51-7.84)	< 0.001
Children 12-24 months	487	427	533	432	490	456				
Measles immunisation received	415 (85.2)	374 (87.6)	422 (79.2)	315 (72.9)	421 (85.9)	382 (83.8)	1.95 (1.08-3.51)	0.027	3.16 (1.77-5.63)	< 0.001
Uptake Of Nutrition Services, n (%)										
Children 0-36 months	1263	1245	1360	1130	1256	1177				
Regular food received from AWW in last 3 months	1030 (75.7)	946 (76.0)	954 (75.5)	897 (79.4)	854 (68.0)	913 (77.6)	0.32 (0.24-0.45)	< 0.001	0.67 (0.48-0.91)	0.012
Child weighed by AWW at least once in the last 3 months	811 (64.2)	901 (72.4)	937 (68.9)	662 (58.6)	809 (64.4)	623 (52.9)	0.80 (0.61-1.04)	0.093	0.66 (0.50-0.86)	0.002
Children 6-36 months	1092	1081	1215	956	1073	1048				
Children aged 6-36 months with SAM (WHZ < -3SD)	61 (5.6)	98 (9.1)	78 (6.4)	91 (9.5)	77 (7.2)	80 (7.6)	0.47 (0.29-0.76)	0.002	0.72 (0.44-1.16)	0.177
Children aged 6-36 months with SAM referred to Nutrition Rehabilitation Centre	1 (1.6)	25 (25.5)	11 (14.1)	8 (8.8)	6 (7.8)	15 (18.8)	0.06 (0.005-0.75)	0.029	0.24 (0.02-2.98)	0.269

^a^Adjusted for baseline differences in anthropometry, tribal/caste status (categorical), ownership of MNREGA card (binary), any maternal education (binary), asset quintile (categorical), district (categorical, fixed effect) and clustering (random effect)

**Fig. 3:**
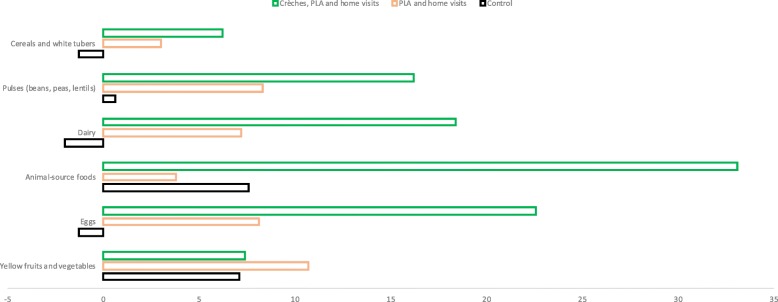
Change in the proportion of children aged 6–36 months consuming each of six food groups, by study area (authors’ own)

Being in areas 2 or 3 was not associated with changes in the timely introduction of complementary feeding, though numbers in the 6–9 months age group were small. Figure 3 shows the change in the proportion of children aged 6–36 months consuming each of six food groups. In area 3, we found increases in the consumption of protein-rich foods including pulses and nuts, animal-source foods and eggs.

Children in areas 2 and 3 were less likely to receive food from *Anganwadi* workers and less likely to be weighed by them in area 3. We found a significant reduction in the proportion of children with Severe Acute Malnutrition in area 2 but no significant reduction in area 3, as consistent with our overall results for wasting.

### Costs

The total and average annual costs of all interventions were USD 1,415,816 and USD 471,939 in area 3 and USD 500,931 and USD 166,977 in area 2, respectively. The estimated average annual costs of the intervention per child under three were USD 82 in area 3 and USD 7.5 in area 2. If one considers beneficiaries to be all those living in the study areas, the average annual costs of the intervention were USD 5.5 and USD 0.5 per person covered in area 3 and area 2, respectively.

## Discussion

Our quasi-experimental study found reductions in the prevalence of wasting, underweight and stunting among children under three in areas with crèches, PLA meetings and home visits. We also found reductions in the prevalence of wasting and underweight in areas with PLA meetings and home visits only. Effects in both areas were greatest among the most marginalised children. Further analyses of continuous z scores confirmed significant effects on WAZ and HAZ in area 3, and smaller, non-significant effects on WHZ and WAZ in area 2. There were significant improvements in several but not all infant feeding and infection control practices in both intervention areas (with and without crèches).

How do the effects of AAM interventions compare with those of other strategies? Global systematic reviews of breastfeeding promotion, complementary feeding education and food supplementation suggest that these can have small to moderate effects on children’s nutritional status, depending on background levels of food security [17–19]. This has been confirmed in several Indian studies. In Haryana and Andhra Pradesh, trials of complementary and/or responsive feeding education found no or small differences in weight and length in children below 2 years [20, 21]. In Jharkhand and Odisha, a trial testing the effects of PLA meetings with women’s groups and counselling through home visits found increases in women’s and children’s dietary diversity, a reduction in underweight and reduced infant mortality, but no significant reductions in wasting or stunting [11].

The existing evidence confirms that interventions to improve infant and young child feeding with no additional food or micronutrient supplementation will have only modest effects on children’s nutritional status in food insecure areas, and must be combined with interventions to control infections, enhance children’s diets, and improve women’s health and nutrition in the prenatal period [22]. This recommendation is supported by data from longitudinal cohorts and modelling studies, which found that between 20 and 30% of cases of stunting and wasting are attributable to foetal growth restriction, highlighting the need to intervene before and during pregnancy [23–26].

The Government’s Integrated Child Development Services focus on providing food supplementation to pregnant women and children, but this remains insufficient to stem the tide of undernutrition. Crèches can provide additional supplementation and co-benefits for caregivers and children in underserved areas. Our study is one of only three experimental and quasi-experimental evaluations of crèches from India to date. A recently completed trial from rural Udaipur, Rajasthan, found a small (4%) reduction in the prevalence in child wasting 1 year after the introduction of affordable day care centres for children aged one to six years [27]. A quasi-experimental study from Dhar and Singrauli districts, Madhya Pradesh, evaluated the conversion of 5% of Anganwadi Centres (AWC) into AWC-cum-crèches. There was limited government investment in crèches and only basic additional training for Anganwadi workers. As a result, only one in 12 children under three years attended crèches, and there were no detectable effects on children’s nutritional status [28]. The utilisation of crèches in AAM was more comparable to that in the Rajasthan study, where over 40% of caregivers of children in the eligible age range ever used crèches. We found larger effects on nutritional status than the Rajasthan study; this may be because our endline survey took place after 3 years of implementation vs. after 1 year only in Rajasthan.

The effects seen on the prevalence of stunting and continuous HAZ in areas with crèches may be explained by the benefits of safe water and a smoke free environment, children’s handwashing routine, their long exposure to crèches if they attended from 6 months to 3 years, and the increased consumption of protein-rich foods, including eggs. A recent trial of egg supplementation once a day for 6 months in children aged 6–9 months in Ecuador found that eggs increased length-for-age z score by 0.63 (95 CI: 0.38–0.88) and WAZ by 0.61 (95% CI: 0.45–0.77) [29].

How can we explain that significant effects were found on the prevalence of wasting and underweight in area 2, but not on continuous z scores? We think there are three possible reasons for this: interventions may have averted some cases of wasting without significantly changing the overall distribution of WHZ; our adjusted difference-in-difference analysis may have had different implications for binary and continuous variables; differences in wasting may have been due to chance or measurement error. We are more confident about the effects of interventions on underweight, as we found fewer WAZ values flagged as implausible in our dataset than HAZ or WHZ values. Nevertheless, the trends of increase in WAZ and WHZ in area 2 are consistent with the reductions in underweight and wasting, and effects on WAZ and HAZ in area 3 were strongly significant.

The effects of AAM interventions among the most marginalised children merit further explanation. The AAM initiative selected poor districts within poor states, and provided a universal opportunity to participate voluntarily in meetings that used visual materials and stories resonating with the poorest mothers, as well as free, universal access to crèches with extra support for children with growth faltering. The objective was to shift the distribution of risk within a population rather than simply targeting high-risk individuals [30]. The combination of universal access to interventions with a preferential option for the most disadvantaged may have helped achieve equitable effects.

The cost of running crèches was higher than that of other AAM activities because of infrastructure and supplementation costs, and because crèches’ extra support for monitoring, supervision and research was included as indirect programme cost. As crèche programmes grow and run to full capacity, one might expect the costs per child to come down. Crèches may also provide several co-benefits: more mothers may be able to rest, engage in income-generation activities, and reduce their expenditure on food and care. Another substantial benefit may arise through long-term gains in children’s development. A 2012 systematic review found large, positive effects of crèches on child development outcomes, but with no studies from India [28]. A recent effectiveness study of low-coverage government crèches in Madhya Pradesh did not find any effects on children’s cognitive ability, suggesting that crèches may need increased population coverage and focused attention on stimulation for these to occur [31].

Our quasi-experimental study had several strengths. It is one of only three studies that attempted to quantify the potential contribution of crèches to reducing undernutrition among children under 3 years in India. It also benefitted from a concurrent control area. The study also had limitations. First, the selection of districts, blocks and clusters in each state was purposive, and we were unable to include data from Bihar and Chhattisgarh. Although this limits the generalisability of our findings, data from Jharkhand and Odisha provide evidence for other, similar areas with high levels of child undernutrition. Second, after two attempted visits, we could interview around 75% of eligible respondents in the endline survey; we may therefore have missed more vulnerable mothers who migrated or worked for substantial periods of time outside their homes. Third, it is possible that mothers’ responses to questions about behaviours were influenced by a desire to give desirable answers following exposure to the interventions or routine ICDS activities. This would have led us to overestimate the benefit of interventions for feeding and infection control practices. Finally, we did not randomise allocation to the different interventions, and deliberately selected poorer areas with more tribal families to establish crèches. We therefore cannot rule out effects of selection bias as well as seasonal and secular changes, although our adjustments for variables that differed by area at baseline, difference in difference analyses, and the fact that baseline and endline surveys were carried out in the same season all attempted to mitigate this.

What do our findings mean for policy and practice? The interventions tested in this study could be scaled up through several pathways. Since 2018, the National Health Mission recommends home visits by ASHAs to improve the care of infants and young children. ASHAs receive 3 days of training and incentives to visit caregivers at home 3, 6, 9, 12 and 15 months after birth. During these visits, they counsel caregivers on responsive feeding, immunisations, as well as play and communication for early childhood development [32]. Our study supports the use of such visits, and suggests that encouraging caregivers to join local groups or visit crèches (where available) could also benefit children whose growth is faltering. Various strategies for scaling up group interventions to improve maternal and child health and nutrition are also being tried out. Some involve ‘participatory communication’ (i.e. health messages imparted through participatory activities) during brief sessions with existing Self-Help Groups; there is some evidence of effects of such approaches on self-reported antenatal, delivery and postnatal behaviours, but no demonstrated effects on mortality or anthropometric outcomes yet [33]. Other strategies, such as PLA, include participants both within and beyond SHGs, and involve broader community mobilisation with participatory problem prioritisation and solving; PLA has been shown to reduce neonatal and infant mortality, as well as underweight in infants and young children [6, 7, 11]. India’s National Health Mission’s and WHO have endorsed PLA as a scalable community mobilisation approach to improve maternal, newborn and child health, and the NHM have already integrated IYCF into its recommended cycle of PLA meetings with groups for ASHA facilitators and ASHAs, providing a clear pathway for scale up [34, 35]. Finally, our data support the National Nutrition Strategy’s call for converting 5% of Anganwadi Centres into AWC-cum-crèches [36]. Several civil society organisations in Jharkhand, Odisha, Chhattisgarh, Madhya Pradesh, Delhi and Rajasthan have now shown the feasibility of setting up of quality crèches in underserved districts, showcasing possible models for government to take up or support. A new mechanism to fund crèches also exists in underserved areas: District Mineral Foundations (DMF) were set up in 2015 using royalties from mining companies. DMF are state-based, non-profit, government-managed trusts created to invest in education and health for communities affected by mining, many of which have high rates of child undernutrition. DMF and other related funds could support further scale up of crèches in partnership with civil society organisations [37]. There are signs that crèches are being mainstreamed through DMF in Odisha and Madhya Pradesh, through the government’s own Phulwari programme in Chhattisgarh, as well as for Particularly Vulnerable Tribal groups in districts of Odisha. These initiatives provide encouraging signals of support for scale up. We are able to provide simple protocols for crèches that cater to undernourished children with mostly local resources.

Future research on integrated community interventions such as AAM should assess their cost-effectiveness, effects on children’s development, maternal mental health, and household expenditure as key potential co-benefits of these interventions.

## Conclusion

Crèches, monthly PLA meetings and counselling through home visits reduced undernutrition among children under three in rural districts of eastern India. These interventions could be scaled up through government’s existing plans for home visits and community mobilisation with ASHAs and ASHA facilitators, and through efforts to support crèches.

## Additional files

Additional file 1: Action Against Malnutrition Survey. (PDF 308 kb)

Additional file 2: Effects of interventions on children’s weight-for-height, weight-for-age and height-for-age z scores. (PDF 34 kb)

Additional file 3: Dataset. (CSV 3605 kb)

## Data Availability

The survey questionnaire is included as Additional file 1. A .csv file containing data and variables used in the analyses presented in this article is attached as Additional file 3.

## Abbreviations

AAM: Action Against Malnutrition
ASHA: Accredited Social Health Activist
CINI: Child In Need Institute
HAZ: Height-for-Age z score
ICDS: Integrated Child Development Services
INR: Indian Rupees
IYCF: Infant and Young Child Services
MAA: Mother’s Absolute Affection
MCP: Mother and Child Protection
MGNREGA: Mahatma Gandhi National Rural Employment Guarantee Act
MUAC: Mid-Upper Arm Circumference
OR: Odds Ratio
PHRN: Public Health Resource Network
PHRS: Public Health Resource System
PLA: Participatory Learning and Action
SAM: Severe Acute Malnutrition
SD: Standard Deviation
WAZ: Weight-for-Age z score
WHZ: Weight-for-Height z score
