# Supplementary material for: Effects of participatory learning and action with women’s groups, counselling through home visits and crèches on undernutrition among children under three years in eastern India: a quasi-experimental study
Source: BMC Public Health. 2019 Jul 18;19:962. doi: 10.1186/s12889-019-7274-3 (PMC6637592; doi:10.1186/s12889-019-7274-3)
Supplement: Supplementary file 1 — Action Against Malnutrition Survey. (PDF 308 kb) [file 12889_2019_7274_MOESM1_ESM.pdf]

Action Against Malnutrition Survey

Mother ID							
**Questionnaire ID**							

Informed consent from the Mother:

Namaskar/Johar. My name is ____________________ I work for Action Against Malnutrition Project, and we are conducting a study about the health status of children (under 3) living in Jharkhand, Chattisgarh, Bihar and Odisha.

You should only participate if you want to; choosing not to take part will not disadvantage you in any way. Before you decide whether you want to take part, it is important to listen to the following information carefully and discuss it with others if you wish. Ask us if there is anything that is not clear or if you would like more information.

If you agree to take part we will ask you to help us to complete some general questions about you and your household, and about feeding habits of your family. We would also like have information about your child's health, nutrition and also about any illness your child may have had . Finally, we would like to assess the growth of your child by weighing him and measuring his height and arm circumference. We will give you feedback about your child's measurements and explain what they mean.
When we take measurements of your child we may find out that if they are small and/or thin for their age and they could benefit from some extra help.

When we have analysed the data we will disseminate the findings to you.

Some information we collect will make it possible to identify you as a participant in this study. Any personal identifiers that could reveal your identity will be removed before the results of the study are made public or shared between people other than the main researchers working on the project. Neither the researcher will speak about anything that you personally have said unless you indicate that there is a real risk to your own or your child's health.

This interview will take 1 hour to complete. Your participation is voluntary and you can chose to discontinue your participation at any time without giving a reason.

We thank you for taking time to understand and your interest in the study.

Interview procedure and process explained ? Yes No

Consent given? Yes No

Signature / Thumb print (primary respondent)___________________________

[***If consent is given proceed with the interview, if consent is refused close the interview***
Interviewer's notes: Please write your own comments on the following points: Any problems in arranging the interview (e.g. finding the respondent / getting respondent to agree to the interview / respondent having time to do interview) or conducting the interview)
____

Any other observations: ____

Block	
Panchayat	
Village name	
Hamlet	
Household identification note	
Name of monitor/interviewer	

SECTION 1: BACKGROUND INFORMATION				
**No.**	**Question**	**Response**	**Code**	**Go to**
1_01	District	West Singhbhum	1	
		Mayurbhanj	2	
		Keonjhar	3	
		Ranchi	4	
		Ramgarh	5	
		Sarguja	6	
		Motihari	7	
1_02	Cluster code			
1_03	Name of Mother	____		
1_04	Name of father	____		
1_05	Date of interview	_____/______/______<br>(day) (month) (year)		
1_06	How many living children less than 3 years old, including newborns, do you have? *Number of children* --------	-------------------------		
1_07	Names of all children below the age of 3 years (oldest child first)	Names / Sex / (day) month)(year)		
		1 / / /		
		2 / / /		
		3 / / /		
		4 / / /		
1_08	What is the birth interval between this child and the last living child	……………… in months<br>……………… in years		
		If the mother has only one child/ or this is the first child	-8	
1_09	Did you attend any PLA meetings about children's health issues? (Only for PLA and crèche)	Yes	1	
		No	2	2_01
1_10	How many of these meetings did you attend?	_____________		
1_11	What kind of benefit did you get?<br>(circle all mentioned by mother – do not prompt)<br>*[Multiple response]*	1. Information	1	
		2. Support from other women	2	
		3. Support from facilitator	3	
		4. monetary support	4	
		5. Other---------------------	5	
		6. Other---------------------	6	
		7. Other---------------------	7	
		8. Other---------------------	8	
**SECTION 2: SOCIO-ECONOMIC INFORMATION**				
2_01	Do you belong to any of the following?	Scheduled Tribe (ST)	1	2_02
		Scheduled Caste (SC)	2	2_03
		Other Backward Caste (OBC)	3	2_04
		None of the above	4	2_04
2_02	Which tribe do you belong to? (name of the tribe)	Please specify……………………….		
2_03	Which caste do you belong to? (name of the caste)	Please specify……………………….		
2_04	Highest years of schooling completed by you?	Class………………		
		No schooling	-8	2_06
2_05	Can you read this passage for me please?<br>(Interviewer to decide the ability level)	Easily	1	
		With difficulty	2	
		Cannot read	3	

SECTION 1: BACKGROUND INFORMATION					
**No.**	**Question**	**Response**	**Code**		**Go to**
			Y	N	
2_06	Which of these do you presently have in your household?<br><br>*[Ask for each item]*	1.Mattress	1	2	
		2.Cot / bed	1	2	
		3.A chair	1	2	
		4.A table	1	2	
		5.A pressure cooker	1	2	
		6.Electricity	1	2	
		7.A Fan	1	2	
		8.Radio / Transistor / Tape	1	2	
		9.TV	1	2	
		10.Watch / Clock	1	2	
		11.Telephone / mobile phone	1	2	
		12.Animal-drawn cart	1	2	
		13.Bicycle	1	2	
		14.Motorcycle	1	2	
2_07	Do you have any agricultural land?	Yes	1		
		No	2		
2_08	Do you own a NAREGA card	Yes	1		
		No	2		2_11
2_09	Have you or any of your family members ever worked under NREGA?	Yes	1		
		No	2		2_11
2_10	For how many days did you/your family members work under NREGA in the last one year	…………….. in days			
2_11	What kind of a ration card do you have?	APL<br>BPL<br>AAY<br>No Ration Card	1<br>2<br>3<br>4		
2_12	Did you get rations (rice/wheat) from the ration shop in the last three months (ask for the last three completed months)?	Yes, every month<br>Yes, only once<br>Yes, twice<br>No, not at all	1<br>2<br>3<br>4		

SECTION 3 : HEALTH RELATED - *Questions related to DIARRHOEA*					
3_01	What is the **main** source of drinking water for members of your household? (for **most** of the months of the year)	Hand pump/Tube well	1		
		Dug well (covered)	2		
		Dug well (not covered)	3		
		River/Canal/Stream /Spring	4		
		Chuan/ Surface water/ Pond	5		
		Others............................	6		
3_02	Which water treatment method do you **mostly** use before drinking water	No water treatment	1		
		Boiling	2		
		Chlorine treatment	3		
		Filter with cloth	4		
		Others	5		
3_03	Is soap used in your house	Yes	1		
		No	2		3_05
			Y	N	
3_04	What are the instances in which you use soap to wash your hands?<br><br>*[Ask for each option]*<br>*[Multiple response]*	1. After use of toilet	1	2	
		2.Before eating	1	2	
		3.After attending a child who has defecated	1	2	
		4.Before preparing food	1	2	

		5.Before feeding child	1	2	
		6.After handling cow dung/dirt	1	2	
3_05	How do you handle or dispose of children's faeces **most** of the times?	Children always use the latrine	1		
		Children's faeces are thrown outside	2		
		Children's faeces are thrown into the latrine	3		
		Children's faeces are rinsed away	4		
		Buried /Covered with mud/Ash	5		
		Other_________	6		
3_06	Do you wash vegetables and fruits before consumption?	Always	1		
		Most of the time	2		
		Rarely	3		
		Never	4		
3_07	How do you wash utensils before feeding?	Use soap	1		
		Not used soap	2		
3_08	Have you ever fed your baby with cup/bowl and a spoon	Yes	1		
		No	2		
SECTION RELATED TO ARI determinants					
3_09	In this household, which of the following do you use **most** of the times to cook food?	Stove	1		
		Chullah	2		
		Open Fire	3		
		Other_________	4		
3_10	Does one of your household members (people with whom you live under the same roof) **smoke** cigarettes/bidi/other tobacco?	Yes	1		
		No	2		
		Don't know	-9		
3_11	Where do you **mostly** cook food?<br><br>*Ask and observe at the same time*	In the same room where you sleep in	1		
		In a separate room/kitchen	2		
		Outdoors	3		

SECTION 4 : FOOD SECURITY

No.	Question	Response	Code		Go to
4_01	What is your family's **main** staple food?	Rice	1		
		Wheat	2		
		Ragi	3		
		Other, specify..............................	4		
4_02	What is the main way that you obtain your family's staple food **most** of the time?	Own production	1		
		Purchased from regular shop	2		
		Purchased from ration	3		
		Barter, borrow, exchange for labour, gift	4		
		Other, specify...............................	6		
			**Y**	**N**	
4_03	Has your household experienced any of the following during the **last 12 months**?<br><br>*[Ask for each option]*<br>*[Multiple response]*	1. A major health problem in your household (if different from disease epidemic)	1	2	
		2. Marriage of a family member	1	2	
		3. Damage to houses or crops by elephants	1	2	
		4. Death of a close one	1	2	
		5. Did not experience any such problem in last 12 months	1	2	

	SECTION 5 A: CHILD HEALTH			
**No.**	**Question**	**Response**	**Code**	**Go to**

5_01	Name of the child	____________________________		
5_02	Do you have a vaccination/ immunization/ health card? May I see it?	Yes	1	
		No	2	
5_03	Date of birth of the child<br>(confirm the date of birth from vaccination/ immunization/ health card)	From card: ____/___/____<br><br>From recall: ____/____/____<br><br>Do not know	 <br><br><br>-9	
5_04	Has your baby ever been breastfed?	Yes	1	
		No	2	5_12
5_05	Was anything given to the baby (to drink, lick) immediate after birth?<br>*Probe (pre lacteal feeding)*	Yes<br>No<br>Don't know	1<br>2<br>-9	
5_06	Was colostrum given to the baby (name) immediate after birth?	Yes<br>No<br>Don't know	1<br>2<br>-9	
5_07	When was the baby put to the breast after birth?	Within 1 hour	1	
		After 1 hour	2	
		Within 3 days	3	
		After 3 days	4	
		Not breastfed	5	
		Don't know	-9	
5_08	How old was ***(name)*** the first time you gave him/her something other than breast milk?<br>*(Exclusive Breastfeeding)*	______months<br><br>*(if less than 1 months old, please mention 0)*		
5_09	Is (child's name) still being breastfed?	Yes	1	
		No	2	5_12
5_10	Was your child breastfed in the last 24 hours?	Yes	1	
		No	2	5_12
5_11	How many times was your child breastfed in last 24 hours ?	__________times		
5_12	**Aside from breast milk**, did ***(name)*** have cow/goat milk since this time yesterday?	Yes	1	
		No	2	5_14
5_13	How many times did your child receive cow/goat milk (**NOT breast milk**) in the last 24 hours?	……… times		
5_14	**Aside from breast milk**, did ***(name)*** have infant formula/powdered milk since this time yesterday?	Yes		
		No		5_16
5_15	How many times did your child receive infant formula/powdered milk (**NOT breast milk**) in the last 24 hours?	……… times		
5_16	Has [name] ever been fed anything with a bottle?	Yes	1	
		No	2	5_18
5_17	Was he/she bottle-fed yesterday	Yes	1	
		No	2	

			**Y**	**N**	**DK**	
5_18	Now I would like to ask you about other liquids that (name) may have had **since this time yesterday** during the day or at night. I am interested in whether your child had the item even if it was combined with other food or drink.<br><br>(Did (name) drink/eat any of the following?)<br>*[Multiple response]*	1. Vitamin or mineral syrups	1	2	-9	
		2. Iron syrup	1	2	-9	
		3. ORS	1	2	-9	
		4. Plain water	1	2	-9	
		5. Infant formula	1	2	-9	
		6. Milk such as tinned /powdered/ fresh animal milk	1	2	-9	

	[Ask for each option]	7. Clear broth (*maad pani*)	1	2	-9	
		8. Yogurt (*dahi*)	1	2	-9	
		9.Thin porridge(*daliya*)	1	2	-9	
		10.Any other liquid (specify)____	1	2	-9	
5_19	Other than the liquids mentioned above, does ___ (NAME) normally eat/consume any solid/semi-solid food?	Yes<br>No<br>Dont Know		1<br>2<br>-9		
5_20	How old was ___(NAME) when he/she was first fed any solid/semi-solid food	________ months<br>Not yet fed		-9		
			**Y**	**N**	**DK**	
5_21	So, since this time yesterday, did ***<u>(name)</u>*** drink/eat at least 10g of the following:<br><br>**WILL NEED TO SHOW GM WHAT 10G LOOKS/FEELS LIKE OF DIFFERENT COMMON FOODS**<br><br>*[Multiple response]*<br>*[Ask for each option]*	1. Bread, Rice, Wheat or other foods made from grains, including thick grain-based porridge?	1	2	-9	
		2. Pumpkin, carrots, or sweet potatoes that are yellow or orange inside?	1	2	-9	
		3. White potatoes, white yams or any other foods made from roots?	1	2	-9	
		4. Any dark green leafy vegetables?	1	2	-9	
		5. RIPE mangoes, RIPE papayas or *(insert other local vitamin A-rich fruits)*?	1	2	-9	
		6. Any other fruits or vegetables? (Including UNRIPE mangoes and papayas and locally grown fruits)	1	2	-9	
		7. Liver, kidney, heart or other organ meats?	1	2	-9	
		8. Any meat, such as beef, pork, lamb, goat, chicken, or duck?	1	2	-9	
		9. Eggs?	1	2	-9	
		10. Fresh or dried fish, shellfish, or seafood?	1	2	-9	
		11. Any foods made from beans, peas, lentils, or nuts?	1	2	-9	
		12. Cheese, yogurt, or other milk products?	1	2	-9	
		13. Any oil, fats, Ghee, butter or foods made with any of these?	1	2	-9	
		14. Any sugary foods such as sweets, candies, cakes or biscuits?	1	2	-9	
		15. Condiments for flavour, such as chillies, spices, herbs or fish powder?	1	2	-9	
		16. Grubs, snails or insects?	1	2	-9	
		17. Foods made with red palm oil, red palm nut, red palm nut pulp sauce?	1	2	-9	

		18. Any other food?	1	2	-9	
5_22	How many times did your child receive semi solid, mushy, or solid feeds (**NOT breast milk**) in the last 24 hours?	_________times				

SECTION 5B: [NAME'S] VACCINATIONS & HEALTH						
**No.**	**Question**					
**Look at all the records available for the child and speak to the mother to try to fill in the following**						
		**YES from card**	**YES from recall**	**NO**	**DK**	
5_23	Measles *(specify between 9 months to 1 year)*	1	2	0	-9	
5_24	Other specify (*Hepatitis or others)*	1	2	0	-9	
5_25	Has (name) taken any drug to get rid of intestinal worms in the past 6 months?	1	2	0	-9	
5_26	Has [name] received vitamin A solution in the past 6 months?	1	2	0	-9	
	**Knowledge section**					
5_27	How much drink should be given to a child during diarrhoea?	Less than usual			1	
		Same as usual			2	
		More than usual			3	
		Not given anything to drink			-8	
5_28	How much a child should be given to eat during diarrhoea?	Less than usual			1	
		Same as usual			2	
		More than usual			3	
		Not given anything to eat			-8	
5_29	How much breastfeed should be given ***to a child*** during diarrhoea?	Less than usual			1	
		Same as usual			2	
		More than usual			3	
		Not breastfed at all			-8	
5_30	How much drink should be given to a child during fever and cough?	Less than usual			1	
		Same as usual			2	
		More than usual			3	
		Not given anything to drink			-8	
5_31	How much a child should be given to eat during fever and cough?	Less than usual			1	
		Same as usual			2	
		More than usual			3	
		Not given anything to eat			-8	
5_32	How much breastfeed should be given ***to a child*** during fever and cough?	Less than usual			1	
		Same as usual			2	
		More than usual			3	
		Not breastfed at all			-8	
5_33	Has (name) had diarrhoea (**loose stools more than 3 times a day)** in the last 2 weeks**?**	Yes			1	
		No			2	5_40
5_34	How much (name) was given ***to drink*** during the diarrhoea?	Less than usual			1	
		Same as usual			2	
		More than usual			3	
		Not given anything to drink			-8	
5_35	How much (name) was given ***to eat*** during the diarrhoea?	Less than usual			1	
		Same as usual			2	
		More than usual			3	
		Not given anything to eat			-8	
5_36	How much breastfeed was given ***to (name)*** during the diarrhoea?	Less than usual			1	
		Same as usual			2	

		More than usual	3	
		Not breastfed at all	-8	
5_37	Did you **seek advice** about the diarrhoea from any source?	Yes	1	
		No	2	5_39
5_38	From whom did you first seek **advice** about the diarrhoea?	AWW	1	
		ASHA	2	
		ANM	3	
		Women's group member	4	
		Creche worker	5	
		Other __________________	6	
5_39	Was he/she given any of the following at any time since he/she started having the diarrhoea	A fluid made from a special packet called ORS?	1 2	
		Gruel made from rice [other local grain]?	1 2	
		Homemade ORS (*namak cheeni ka ghol*)	1 2	
		Nothing was given	1 2	
		Other___________________	1 2	
5_40	Has (name) been ill <u>with a fever and cough</u> at any time <u>**in the last 2 weeks?**</u>	Yes	1	
		No	2	5_46
5_41	How much (name) was given ***to drink*** during the fever and cough?	Less than usual	1	
		Same as usual	2	
		More than usual	3	
		Not given anything to drink	-8	
5_42	How much (name) was given ***to eat*** during the fever and cough?	Less than usual	1	
		Same as usual	2	
		More than usual	3	
		Not given anything to eat	-8	
5_43	How much breastfeed was given ***to (name)*** during the fever and cough?	Less than usual	1	
		Same as usual	2	
		More than usual	3	
		Not breastfed at all	-8	
5_44	Did you seek **advice** about the fever and cough from any source?	Yes	1	
		No	2	5_46
5_45	From whom did you first seek **advice** about the fever and cough?	AWW	1	
		ASHA	2	
		ANM	3	
		Women's group member	4	
		Creche worker	5	
		Other specify…………….	6	

			Y	N	DK	
5_46	Have you used any of these services of the Anganwadi Center in last 3 months?<br><br>*[Multiple response]*<br>*[Ask for each option]*	1. Take-home rations for women and infants	1	2	-9	
		2. Immunization	1	2	-9	
		3. Health check up for children	1	2	-9	
		4. Growth monitoring of children (child weighed)	1	2	-9	
		5. Health referral services	1	2	-9	
		6. Health and nutrition counselling for parents	1	2	-9	
		7. Home visits by Anganwadi worker	1	2	-9	
5_47	<u>**If child is 6 months or older:**</u><br>In the **last 3 months** how often has (NAME) received	Regular			1	
		Irregular			2	

	food from the Anganwadi worker?	Never received	3	

5_48	In the **last 3 months** how many times has [name's] weight been measured by the AWW?	Not at all	-8	
		At least once a month	1	
		At least once in 3 months	2	
		Any other.........................	3	
		Don't know	-9	
5_49	After [name] was weighed from whom did you receive nutritional counselling?	None	-8	
		Anganwadi worker	1	
		ASHA	2	
		ANM	3	
		Womens' group facilitator	4	
		Group member	5	
		Others.................	6	
5_50	Was your child referred to MTC or NHRC ever?	Yes	1	
		No	2	
5_51	How many times in total in the ***last 3 months*** has your child been ill/sick?	None	-8	
		1-2 times	1	
		3-4 times	2	
		5-6 times	3	
		7 or more times	4	
		Don't know/can't remember	-9	

**Questions Related to Malaria**				
5_52	Do you have a mosquito net in the house?	Yes	1	
		No	2	5_55
5_53	What kind of net did (name) sleep under last night?	Ordinary net	1	5_55
		Insecticide treated bed nets(ITBN)	2	5_55
		Did not use a bed net	3	
5_54	Why did (name) not sleep under a bed net?	Did not see a need to use a net	1	
		Does not feel comfortable sleeping under a bed net	2	
		Less or no mosquito bites in our locality	3	
		Baby was covered with cloth last night	4	
		Bed net damaged	5	
		Any other_____________	6	
5_55	In the last 3 months did the child [name] have a blood test for malaria?	Yes	1	
		No	2	6_01
		Do not know	-9	6_01
5_56	Was the baby [name] treated for malaria **(not with herbal medicines)**?	Yes	1	
		No	2	
		Do not know	-9	

SECTION 6: ANTHROPOMETRY

<table>
<tr><th>No.</th><th>Question</th><th>Response</th><th>Code</th></tr>
<tr><td>6_01</td><td>Name of the child</td><td></td><td></td></tr>
<tr><td>6_02</td><td>Sex of the child Male
Male
Female</td><td>
1
2</td><td></td></tr>
<tr><td>6_03</td><td>Age of the child in months</td><td>----------in months

----------in years</td><td></td></tr>
<tr><td>6_04a

6_04b</td><td>Weight of the child: 1st ___ ___ . ___ ___ Kg

Weight of the child: 1st ___ ___ . ___ ___ Kg</td><td><table><tr><th></th><th>Weight of the mother</th><th>Weight of mother and child</th></tr><tr><td>1</td><td></td><td></td></tr><tr><td>2</td><td></td><td></td></tr></table></td><td></td></tr>
<tr><td>

6_05a
6_05b</td><td><u>If child is under 2 years of age or is not able to stand:</u>
Recumbent length of child (in cm to nearest 5mm)
Recumbent length of child (in cm to nearest 5mm)</td><td>__ ___ ___ .___ Cm

__ ___ ___ .___ Cm</td><td></td></tr>
<tr><td>6_06</td><td>Height of child (in cm to nearest 5mm)</td><td>__ ___ ___ .___ Cm</td><td></td></tr>
<tr><td>6_07a

6_07b</td><td>Child MUAC measurement (in cm to nearest mm)

Child MUAC measurement (in cm to nearest mm)</td><td>___ ___ . ___ Cm

___ ___ . ___ Cm</td><td></td></tr>
<tr><td>6_08</td><td>Child MUAC colour category</td><td>Green ☐

Yellow ☐

Red ☐</td><td>1

2

3</td></tr>
<tr><td>6_09</td><td>Child with pitting oedema in BOTH feet?</td><td>Yes
No</td><td>1
2</td></tr>
</table>

Thank you
