# Supplementary material for: Effects of participatory learning and action with women’s groups, counselling through home visits and crèches on undernutrition among children under three years in eastern India: a quasi-experimental study
Source: BMC Public Health. 2019 Jul 18;19:962. doi: 10.1186/s12889-019-7274-3 (PMC6637592; doi:10.1186/s12889-019-7274-3)
Supplement: Supplementary file 2 — Effects of interventions on children’s weight-for-height, weight-for-age and height-for-age z scores. (PDF 34 kb) [file 12889_2019_7274_MOESM2_ESM.pdf]

Supplementary File 2: Effects of interventions on children's weight-for-height, weight-for-age and height-for-age z scores

	**Baseline**	**Endline**	**Absolute change in mean z score between baseline and endline**	**Adjusted coefficient (95% CI)[1]**	***p***
**WHZ, mean (SD)**					
Control	-1.09 (1.33)	-1.07 (1.64)	-0.02		
PLA and home visits	-1.27 (1.35)	-1.03 (1.50)	-0.24	0.15 (-0.02-0.31)	0.079
Crèches, PLA and home visits	-1.11 (1.33)	-1.12 (1.29)	+0.01	-0.08 (-0.24-0.09)	0.328
**WAZ, mean (SD)**					
Control	-2.07 (1.43)	-2.06 (1.57)	-0.01		
PLA and home visits	-2.09 (1.31)	-1.95 (1.34)	-0.14	0.14 (-0.01-0.30)	0.082
Crèches, PLA and home visits	-2.09 (1.40)	-1.92 (1.27)	-0.17	0.18 (0.02-0.33)	0.026
**HAZ, mean (SD)**					
Control	-2.1 (1.78)	-2.01 (1.96)	+0.04		
PLA and home visits	-2.0 (1.73)	-2.06 (1.69)	-0.06	-0.11 (-0.31-0.09)	0.278
Crèches, PLA and home visits	-2.26 (1.73)	-1.96 (1.53)	+0.30	0.25 (0.05-0.46)	0.014

[1] Adjusted for baseline differences in anthropometry, child age in months (continuous), tribal/caste status (categorical), ownership of MNREGA card (binary), any maternal education (binary), asset quintile (categorical), district (categorical, fixed effect) and clustering (random effect).
